# Factors associated with anemia among children in South and Southeast Asia: a multilevel analysis

**DOI:** 10.1186/s12889-023-15265-y

**Published:** 2023-02-15

**Authors:** Dev Ram Sunuwar, Devendra Raj Singh, Pranil Man Singh Pradhan, Vintuna Shrestha, Pushpa Rai, Sunil Kumar Shah, Bipin Adhikari

**Affiliations:** 1 Department of Nutrition and Dietetics, Nepal Armed Police Force Hospital, Kathmandu, Nepal; 2 School of Human and Health Sciences, University of Huddersfield, Huddersfield, UK; 3 Research and Innovation Section, Southeast Asia Development Action Network (SADAN), Lalitpur, Nepal; 4 Department of Community Medicine, Maharajgunj Medical Campus, Institute of Medicine, Tribhuvan University, Kathmandu, Nepal; 5 Nepalese Society of Community Medicine, Kathmandu, Nepal; 6 Dhaulagiri Prabidhik Shikshya Pratisthan, CTEVT, Baglung, Nepal; 7 Department of Nursing, Patan Academy of Health Sciences (PAHS), Lalitpur, Nepal; 8 Public Health and Nutrition Section, Bagmati Welfare Society Nepal, Sarlahi, Nepal; 9 Mahidol-Oxford Tropical Medicine Research Unit, Faculty of Tropical Medicine, Mahidol University, Bangkok, Thailand

**Keywords:** Multilevel analysis, Associated factors, Childhood anemia, South and Southeast Asian countries

## Abstract

**Background:**

South and Southeast Asian countries (SSEA) account for the highest burden of anemia globally, nonetheless, progress towards the decline of anemia has almost been stalled. This study aimed to explore the individual and community- level factors associated with childhood anemia across the six selected SSEA countries.

**Methods:**

Demographic and Health Surveys of SSEA countries (Bangladesh, Cambodia, India, Maldives, Myanmar, and Nepal) conducted between 2011 and 2016 were analyzed. A total of 167,017 children aged 6–59 months were included in the analysis. Multivariable multilevel logistic regression analysis was used to identify independent predictors of anemia.

**Results:**

The combined prevalence of childhood anemia across six SSEA countries was 57.3% (95% CI: 56.9–57.7%). At the individual level, childhood anemia was significantly higher among (1) mothers with anemia compared to non-anemic mothers (Bangladesh: aOR = 1.66, Cambodia: aOR = 1.56, India: aOR = 1.62, Maldives: aOR = 1.44, Myanmar: aOR = 1.59, and Nepal: aOR = 1.71); (2) children with a history of fever in the last two weeks compared to those without a history of fever (Cambodia: aOR = 1.29, India: aOR = 1.03, Myanmar: aOR = 1.08), and; (3) stunted children compared to those who were not (Bangladesh: aOR = 1.33, Cambodia: aOR = 1.42, India: aOR = 1.29, and Nepal: aOR = 1.27). In terms of community-level factors, children with mothers in communities with a high percentage of community maternal anemia had higher odds of childhood anemia in all countries (Bangladesh: aOR = 1.21, Cambodia: aOR = 1.31, India: aOR = 1.72, Maldives: aOR = 1.35, Myanmar: aOR = 1.33, and Nepal: aOR = 1.72).

**Conclusion:**

Children with anemic mothers and stunted growth were found vulnerable to developing childhood anemia. Individual and community-level factors identified in this study can be considered to develop effective anemia control and prevention strategies.

**Supplementary Information:**

The online version contains supplementary material available at 10.1186/s12889-023-15265-y.

## Introduction

Anemia is a significant nutrition problem affecting millions of women and children in developing countries [1], and is characterized by a reduction in hemoglobin concentration, decreased quantity of red blood cells, and subsequent impairment in meeting the oxygen demands of tissues [2, 3]. Anemia affects cognitive development, school performance, physical activity, motor, behavioral growth, and immune function against diseases in young children [4–7] with substantial economic implications [8]. During infancy and pregnancy, demands for iron is accentuated to meet the physiological requirement of oxygen by the growing tissues [2]. During pregnancy, iron demand rises from 0.8 mg per day in the first trimester to 7.5 mg per day in the third trimester [9]. The balance between the supply and demand of iron from mother to child determines the vulnerability of infants to iron deficiency anemia and its consequences [10].

Anemia remains a major cause of morbidity and mortality among children in low and middle-income countries (LMICs) with an estimated 293.1 million (approximately 43% of children) anemic children under five years of age globally [11]. A higher prevalence was shown in the South Asian region with 55.12% in 2016 [12] and 52% in 2019 [13]. Anemia is considered a severe public health problem when the prevalence rate is ≥ 40% in a vulnerable population based on the criteria set by WHO [11]. Young children have the highest prevalence of anemia in all regions [14], the prevalence was reported to be greater than 40% in 34 LMICs in the year 2018 [15].

The causes of anemia can be broadly classified into three major groups: nutritional deficiencies, infectious diseases, and genetic hemoglobin disorders [11]. Nutritional anemia is the major cause [2] particularly for young children as they require high iron quantity to maintain growth [6]. About 50% of anemia cases are due to iron deficiency [16] which results from an insufficient diet [17], inadequate hemopoietic nutrients (iron, vitamin B12, and folic acid) [18, 19], increase intake of absorption enhancers such as vitamin C, phytates (whole grains, legumes), and calcium (dairy products) and increased intake of tea, coffee, and some spices which are known to inhibit the iron absorption [20].

In addition, in LMICs, infectious diseases contribute to anemia through impaired absorption and metabolism of iron. For instance, soil-transmitted hookworms (Necator americanus and Ancylostoma duodenale) are a common cause of anemia in Southeast Asia and Sub-Saharan Africa, with an estimated 576–740 million infections [2]. Likewise, maternal co-morbidities such as vitamin A deficiency, riboflavin, B12, and folate deficiency, sickle cell disease, Glucose-6-phosphate dehydrogenase (G6PD) deficiency, malaria, HIV, and tuberculosis are significant factors in the development of childhood anemia [3]. Prevalence of anemia also varies by socioeconomic factors such as education, household wealth status, occupation, wealth index of communities [2, 21], age, sex, maternal age [5, 22], maternal anemia [5, 23], malnutrition (especially stunting) [5, 22], insufficient meals per day, recent diarrhea, fever, and worm infestations [5, 23, 24].

Despite the implementation of anemia control programs including iron-folic acid supplementation, deworming, and insecticide-treated bed net distribution, South Asian countries account for the largest burden of anemia cases, and progress to decline is almost stalled [21, 25]. Considering the slow progress in the reduction of anemia prevalence, these conditions pose a significant challenge to policymakers tasked with achieving the WHO global nutrition targets 2025 and nutrition targets of the Sustainable Development Goals-2030 [26]. To abate the prevalence of anemia, it is necessary to generate adequate evidence in terms of the role and contribution of an individual, household, and the community. Exploring the commonalities and differences across the states in the South and South East Asia region can inform the regional policy. Despite that country-level prevalence, trend, and individual factors of anemia among women and children have been well explored in previous studies [6, 7, 25, 27, 28], only a few studies have used multicounty data to explore the factors related to childhood anemia in the region. The main objective of this study was to explore the individual and community-level factors affecting childhood anemia across the South and Southeast region. The empirical evidence generated from this study is expected to support policymakers to identify the priority areas and tailoring the interventions at the national and regional levels.

## Methods

### Data sources

This study utilized the data from the DHS of six selected countries in South and Southeast Asia (SSEA) conducted between 2011 and 2016 (Bangladesh DHS 2011, Cambodia DHS 2014, India NFHS 2016, Maldives DHS 2016, Myanmar DHS 2015, and Nepal DHS 2016). Although Bangladesh Demographic and Health Survey 2014 and 2017 datasets are publicly available, we used BDHS 2011 data because the information on hemoglobin levels among children was missing in the latest dataset. We excluded the remaining South and Southeast Asian Countries due to a lack of availability of data on anemia among 6–59 months of children. DHS constitutes a large, nationally representative, community-based household surveys which are usually conducted by in-country/local institutions and are funded by the United States Agency for International Development (USAID), with technical assistance from ORC (Opinion Research Corporation) Macro International Inc. Calverton, Maryland, USA [29]. The DHS programme aims to provide reliable indicators about fertility, family planning, health, and nutrition status in developing countries [29].

### Sampling strategy

The final samples of households in DHS were utilized in a multistage cluster sampling design to provide representative estimates for all enumeration areas (EAs). The probability proportional to size (PPS) sampling strategies were used by DHS to include both rural and urban residents, followed by a random selection of households from within the selected clusters or enumeration areas. All surveys included sample weights in the dataset. A more detailed sampling methodology of DHS has been published elsewhere [30–35]. The DHS datasets for the selected countries were obtained from the DHS program website (URL: https://www.dhsprogram.com/data/available-datasets.cfm) after receiving approval to access and download the DHS data file. We analyzed the children’s recode (KR) file to examine individual and community-level factors associated with childhood anemia in six SSEA countries. In this study, information from households that have at least one mother-child pair including information on child Hb level, anthropometry, and covariates from six selected SSEA countries were incorporated into the analysis. The details of the sample size and exclusion criteria for the selection of the mother-child pairs are presented in the S1 Table.

### Outcome variable

According to the WHO, for children aged 6–59 months, any form of anemia was defined as hemoglobin concentration < 11 g/dL [4]. Hemoglobin level was assessed using capillary blood and the HemoCue rapid testing technique in all six SSEA countries. For additional analysis of the outcome variable, the categories of anemia were further dichotomized as ‘anemic’ and ‘not anemic’. Hemoglobin levels of the children and mothers were measured using HemoCue, which is the standard test used in the DHS program. The HemoCue has been recommended for use as an on-the-spot device for determining hemoglobin in resource-poor settings and tests capillary blood from the finger-prick [36]. The detailed process has been shown on the website ( https://dhsprogram.com/topics/Anemia.cfm ). Previous studies have established HemoCue 201 + as the gold standard for venous blood measurements and have noted its great predictability (98.7%) [37] and sensitivity (93%) [38].

### Predictors of childhood anemia

#### Individual-level factors: child, maternal and household-level factors

Maternal and household, and the characteristics related to children were included in the individual-level factors. A total of 11 individual-level factors were included in this study. Child-level factors included child’s sex (male and female), age in months (6–11, 12–23, 24–35, 36–47, and 48–59 months), history of recent fever (occurrence of fever in the past two weeks), history of diarrhea (occurrence of diarrhea in the last two weeks), and childhood nutritional status: stunting, wasting, and underweight. The WHO Multicenter Growth Reference Study Group, 2006 was used to calculate the anthropometric indicators to evaluate the nutritional status of each child [39]. Children suffering from stunting, wasting, and underweight were defined as children with Z-score below minus two standard deviations (< -2SD), for height-for-age (HAZ), weight-for-height (WHZ), and weight-for-age (WAZ) respectively. Maternal and household-level variables included mothers’ age in years (15–24, 25–34, and ≥ 35 years), mothers’ education attainment (no education, primary and secondary and above), wealth quintile, and maternal anemia level. Maternal anemia was categorized as anemic with a hemoglobin level of < 12 g/dL and otherwise non-anemic [4].

### Community-level factors

The seven different factors were included in the community-level factors (place of residence, community maternal anemia, community parity, community wealth, community female education, community safe water access, and community toilet facility). The aggregate continuous community-level predictor variables were constructed by aggregating individual-level characteristics at the community (cluster) level and further categorization of the aggregate variables was done as “high” or “low” based on the distribution of the proportion values calculated for each community [5, 40, 41]. The mean value was used as a cut-off point of the proportion values for the categorization of community-level variables. The community maternal anemia was defined as the mean percentage of women with Hb level less than 12 g/dL. Similarly, community parity was the mean percentage of women with a fertility rate of five children and above. The community wealth was defined as the mean percentage of richness (rich, richest, and above). Likewise, community female education was defined as the mean percentage of women in the community with at least primary education. The community safe water supply was defined as the mean percentage of households with access to an improved water source (piped water into dwelling, piped water to yard/plot, public tap or standpipe, tube well or borehole, protected dug well-protected spring and rainwater) [42]. The community toilet facility was the mean percentage of households with access to improved toilet facility (flush toilet, piped sewer system, septic tank, flush/pour flush to pit latrine, ventilated improved pit latrine, pit latrine with slab, and composting toilet) [42].

### Data analysis

Data were analyzed using Stata/MP version 14.1 (StataCorp LP, College Station, Texas). The svy techniques are a set of commands that take into consideration stratification, clustering, and sample weights in complex survey data. The datasets from six selected SSEA countries were pooled for analysis. For each country, we estimated overall and country-level weighted prevalence rates and their 95% confidence intervals of childhood anemia. The weighted prevalence of anemia among 6–59 months children was examined according to individual-level and community-level factors respectively. Multivariable multilevel logistic regression (MMLR) analysis was performed to estimate the adjusted odds ratio and to estimate the extent of random variations between communities in each country.

Four models were created and were fitted. Model I (empty model) was fitted without predictor variables to test random variability in the intercept and to estimate the intra-class correlation (ICC). Model II examined the effects of individual-level characteristics. Model III examined the effects of community-level variables and Model IV examined the effects of both individual and community-level characteristics simultaneously. We presented only model IV (final model) in this study (Table 3), while Model I, Model II, and Model III are presented in the S2 Table. In the multivariable multilevel logistic regression models, the fixed effects estimate the association between the likelihood of childhood, individual-level, and community-level factors; and are reported as adjusted odds ratios (aOR). Findings were statistically significant if the p-value was < 0.05 at 95% confidence intervals (CIs). The random effects are expressed as ICC and proportional change in variance (PCV) [43]. Models fit were assessed using Akaike information criterion (AIC) and the Bayesian information criterion (BIC) [44, 45]. Nonetheless, these criteria are contested in the literature, and relying on these criteria alone may have inflated the fit of the model. To prevent statistical bias in the multilevel logistic regression model, we examined and reported multi-collinearity among the predictor variables using variation inflation factors (VIF) (S3 Table). In this study, we used “10” as a cut-off value for the maximum level of VIF [46]. All variables with statistically significant associations (p < 0.05) in bivariate analysis were included in the multivariable multilevel regression analysis. Additionally, ArcGIS Desktop version 10.8 was used to generate the map and display the prevalence of childhood anemia by country. The base file of the administrative national and subnational boundaries was obtained from the freely available copyright-free Natural Earth (URL: http://www.naturalearthdata.com/).

**Table 1:** Socio-demographic characteristics of the study participants by country (n = 167,017)

Study variables	Bangladesh (DHS 2011)	Cambodia (DHS 2014)	India (NFHS 2016)	Maldives (DHS 2016/17)	Myanmar (DHS 2015)	Nepal (DHS 2016)
n	N = 1937	N = 3310	155,393	N = 1696	2934	N = 1747
	n (%)	n (%)	n (%)	n (%)	n (%)	n (%)
**Individual-level factors**						
**Child-level factors**						
**Sex of child**						
Male	1016 (52.5)	1695 (50.9)	81,869 (52.9)	885 (52.9)	1498 (50.8)	926 (52.5)
Female	921 (47.5)	1615 (49)	73,524 (47.1)	811 (47)	1436 (49.2)	821 (47.5)
**Child age (months)**						
6–11	214 (10.7)	361 (10.4)	15,181 (9.8)	146 (9.6)	276 (9.1)	164 (9.4)
12–23	405 (21.2)	801 (24.3)	34,207 (22.1)	374 (21.3)	639 (21.6)	410 (23.4)
24–35	393 (19.6)	766 (23.2)	34,907 (22.3)	363 (20.7)	671 (22)	401 (22.5)
36–47	492 (25.3)	706 (22.1)	36,995 (23.7)	441 (25.1)	730 (24.9)	407 (22.9)
48–59	433 (23.0)	676 (19.9)	34,103 (21.9)	372 (23.1)	618 (22.2)	365 (21.2)
**Recent fever**						
No	1162 (61.6)	2393 (70)	134,160 (86.3)	1343 (76)	2354 (82.7)	1358 (77.3)
Yes	775 (38.3)	917 (29.9)	21,120 (13.6)	353 (23.9)	579 (17.2)	389 (22.6)
**Recent diarrhea**			**(n = 155,249)**	**(n = 1695)**		**(n = 1741)**
No	1825 (94.7)	2907 (87)	141,232 (90.8)	1616 (95.5)	2560 (88.7)	1619 (92.6)
Yes	112 (5.2)	401 (13)	14,017 (9.1)	79 (4.4)	374 (11.3)	122 (7.3)
**Children stunted**	**(n = 1900)**	**(n = 3244)**	**(n = 154,743)**	**(n = 1650)**	**(n = 2897)**	**(n = 1741)**
Not stunted	1118 (58.7)	2146 (66.4)	94,365 (60.9)	1407 (84.7)	1669 (69.9)	1089 (62.8)
Stunted	782 (41.2)	1101 (33.5)	60,378 (39.1)	243 (15.2)	928 (30.1)	652 (37.1)
**Children underweight**	**(n = 1900)**	**(n = 3247)**	**(n = 154,743)**	**(n = 1685)**	**(n = 2898)**	**(n = 1746)**
Not underweight	1193 (62.8)	2460 (74.7)	101,288 (64.2)	1431 (84.3)	2333 (79.6)	1258 (72.1)
Underweight	707 (37.2)	787 (25.2)	53,455 (35.8)	254 (15.6)	565 (20.3)	488 (27.9)
**Children wasted**	**(n = 1900)**	**(n = 3247)**	**(n = 154,743)**	**(n = 1644)**	**(n = 2886)**	**(n = 1742)**
Not wasted	1577 (82.8)	2944 (90.6)	124,061 (79.6)	1488 (90.6)	2698 (92.9)	1572 (90.3)
Wasted	323 (17.1)	303 (9.3)	30,682 (20.3)	156 (9.3)	188 (7)	170 (9.6)
**Mothers and household-level factors**						
**Mothers age (years)**						
15–24	916 (48.6)	855 (26.2)	47,015 (32.5)	225 (12.1)	489 (16.5)	716 (40.2)
25–34	822 (41.6)	1892 (56.7)	90,613 (57.8)	1080 (63)	1493 (51.6)	855 (49)
35–49	197 (9.6)	563 (17)	17,765 (9.6)	391 (24.8)	952 (31.8)	176 (10.7)
**Mothers education level**						
No education	348 (18.8)	454 (13.1)	45,698 (28.2)	25 (1.3)	447 (14.5)	571 (33.9)
Primary	622 (32.2)	1685 (54.9)	22,055 (13.7)	360 (19.4)	1355 (48.2)	316 (18.8)
Secondary or above	967 (48.9)	1171 (31.9)	87,640 (58)	1311 (79.1)	1132 (37.1)	860 (47.1)
**Wealth quintile**						
Poorest	422 (23.2)	764 (24)	38,286 (23.7)	512 (23.9)	877 (29.4)	423 (19.7)
Poor	374 (19.9)	624 (20.1)	35,747 (21.4)	510 (24.8)	661 (22.5)	392 (22.5)
Middle	361 (19.12)	540 (19.5)	31,306 (19.8)	488 (24.7)	546 (17.7)	367 (22)
Richer	371 (19.67)	603 (17.5)	27,024 (18.9)	147 (16.1)	508 (17.1)	350 (21.7)
Richest	409 (18.04)	779 (18.7)	23,030 (16)	39 (10.3)	342 (13.2)	215 (13.9)
**Maternal anemia**						
Not anemic	1117 (56.8)	1866 (55.2)	69,861 (43.6)	702 (38.1)	1691 (57.1)	990 (55.5)
Anemic	820 (43.1)	1444 (44.7)	85,532 (56.3)	994 (61.9)	1243 (42.8)	757 (44.4)
**Community-level factors**						
**Place of residence**						
Urban	611 (22.9)	891 (14.1	38,726 (29.1)	102 (25.7)	613 (22.1)	988 (53.4)
Rural	1326 (77.1)	2419 (85.9)	116,667 (70.9)	1594 (74.3)	2321 (77.1)	759 (46.5)
**Community maternal anemia** ^**a**^						
Low	963 (48.1)	1652 (47.2)	74,851 (46.5)	920 (49.9)	1416 (47.4)	935 (50.2)
High	974 (51.9)	1658 (52.7)	80,542 (53.4)	778 (50.1)	1518 (52.5)	812 (49.2)
**Community parity** ^**b**^						
Low	1274 (66.7)	2068 (63.5)	96,893 (66.7)	814 (58.8)	1769 (68.6)	1041 (59.8)
High	663 (33.2)	1242 (36.5)	58,500 (33.2)	882 (41.1)	1165 (31.3)	706 (40.1)
**Community wealth** ^**c**^						
Low	1028 (49.6)	1790 (52.1)	75,191 (50.9)	734 (55.3)	1378 (49.1)	975 (61.2)
High	909 (50.3)	1520 (47.9)	80,202 (49)	962 (44.6)	1556 (50.9)	772 (36.7)
**Community female education** ^**d**^						
Low	833 (45.1)	1905 (62.5)	75,800 (46.7)	710 (39.2)	1626 (58.3)	847 (51.4)
High	1104 (54.8)	1405 (37.4)	79,593 (53.2)	986 (60.7)	1308 (41.6)	900 (48.5)
**Community safe water access** ^**e**^						
Low	1739 (90.9)	-	97,042 (65)	1213 (56.9)	1772 (59.7)	1206 (71.8)
High	198 (9.1)	-	58,351 (34.9)	483 (43.1)	1162 (40.3)	541 (28.1)
**Community toilet facility** ^**f**^						
Low	1073 (54.4)	1716 (48)	79,454 (49.6)	1575 (93.6)	1429 (44)	1237 (63.6)
High	864 (45.5)	1594 (51.9)	75,939 (50.3)	121 (6.3)	1505 (55.9)	510 (36.3)

Frequency (n) are unweighted; percentage (%) are weighted; ^a^mean percent of women with Hb levels less than 12 g/dL; ^b^ mean percent of women with fertility rate of 5 children and above; ^c^ mean percent of households wealth quintiles categorized richer and richest and above; ^d^ mean percent of women with primary education level and above; ^e^mean percent of households with access to improved water source (piped water into dwelling, piped water to yard/plot, public tap or standpipe, tube well or borehole, protected dug well protected spring and rainwater) ^f^ mean percent of household with access to improved toilet facility (flush toilet, piped sewer system, septic tank, flush/pour flush to pit latrine, ventilated improved pit latrine, pit latrine with slab, and composting toilet)

#### Ethical approval

**and consent to participate**.

The DHS program sought and obtained the required ethical approvals from each respective country: National Ethics Committee of the Bangladesh Medical Research Council [30], Cambodian National Ethical Committee for Health Research [31], Ethics Review Board of the International Institute for Population Sciences, Mumbai, India [32], Ethics Review Committee on Medical Research, Ministry of Health, Maldives [33], Ethics Review Committee on Medical Research, Ministry of Health and Sports, Myanmar [34], and Nepal Health Research Council (NHRC), Nepal [35]. We registered and requested access to data from the DHS website (URL: https://www.dhsprogram.com/data/available-datasets.cfm). DHS programs collect data following written informed consent from each individual. All individual identifiers were precluded from the final dataset in this study. The surveys were conducted in accordance with relevant guidelines and regulations.

## Results

### Prevalence of anemia

**Fig. 1:**
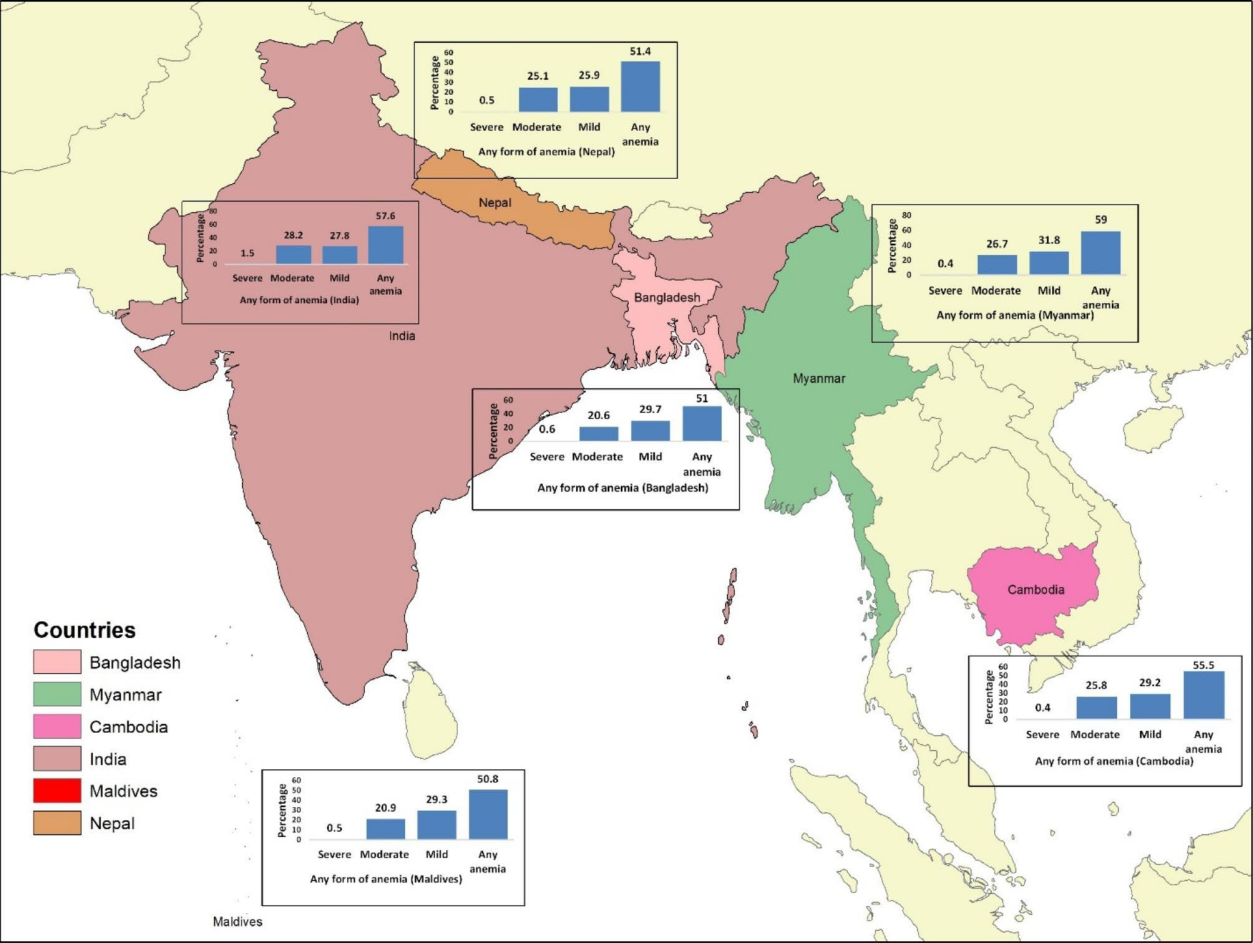
Map showing the prevalence of anemia among children aged 6–59 months in six selected South and Southeast Asian countries (n = 167,017). The map was generated using ArcGIS Desktop version 10.8 (URL: https://desktop.arcgis.com/en/arcmap/)

A total of 167,017 children aged 6–59 months per mother were included in the pooled analysis (Table 1). The overall weighted prevalence of anemia among children in this study was 57.3% (95% CI: 56.9–57.7%), ranging from 50.8% (95% CI: 47.6–53.9%) in the Maldives to 59% (95% CI: 56.6–61.3%) in Myanmar (Table 2). The higher prevalence of mild anemia was 31.8% (95% CI: 29.7–33.9) in Myanmar and the higher prevalence of moderate anemia was 28.4% (95% CI: 27.2–28.6), while the prevalence of severe anemia was less than 1% in most of the countries except India with the prevalence of 1.5% (95% CI: 1.3–1.5) (Fig. 1) (Table 1).

**Table 2:** Bivariate associations between the predictors and childhood anemia (n = 167,017)

	Anemia status %[95% CI]						
Study variables	Bangladesh (DHS 2011)	Cambodia (DHS 2014)	India (NFHS 2016)	Maldives (DHS 2016/17)	Myanmar (DHS 2015)	Nepal (DHS 2016)	SSEA Pooled
Sample sizes (n)	n = 1,937	n = 3,310	n = 155,393	n = 1,696	n = 2,934	n = 1,747	n = 167,017
Overall prevalence	51 (48.4–53.6)	55.4 (53.1–57.7)	57.5 (57.1–57.9)	50.8 (47.6–53.9)	59 (56.6–61.3)	51.4 (48.4–54.3)	57.3 (56.9–57.7)
Mild anemia	29.7 (27.5–32.1)	29.23 (27.3–31.2)	27.8 (27.5–28.1)	29.3 (26.3–32.4)	31.8 (29.7–33.9)	25.9 (23.7–28.1)	27.9 (27.6–28.2)
Moderate anemia	20.6 (18.6–22.8)	25.8 (23.8–27.9)	28.2 (27.2–28.6)	20.9 (17.9–24.3)	26.7 (24.5–29)	25.1 (22.4–27.8)	27.9 (27.6–28.3)
Severe anemia	0.6 (0.3–1.1)	0.4 (0.2–0.6)	1.4 (1.3–1.5)	0.5 (0.2–1.3]	0.4 (0.2–0.7)	0.5 (0.2–0.9)	1.4 (1.3–1.5)
**Individual-level factors**							
**Child-level factors**							
**Sex of child**							
Male	51.7 (48.1–55.3)	57.5* (54.4–60.6)	57.4 (56.8–57.9)	54.4** (50.4–58.2)	58.3 (55.1–61.4)	51.8 (48.1–55.5)	57.2 (56.7–57.7)
Female	50.2 (46.6–53.9)	53.2 (50.1–56.3)	57.7 (57.1–58.3)	46.7 (42.6–50.9)	59.6 (56.5–62.7)	51 (46.6–55.3)	57.4 (56.8–57.9)
**Child age (months)**							
6–11	74.2*** (66.9–80.4)	78.1*** (72-83.2)	67.9*** (66.8–69)	66.2** (55.6–75.3)	79.5*** (72.9–84.8)	69.1** (60.7–76.3)	68.4*** (67.3–69.4)
12–23	68.3 (62.8–73.3)	73.3 (69.3–76.9)	69.6 (68.8–70.3)	53.6 (46.5–60.4)	77.2 (72.7–81.1)	66.1 (60.3–71.3)	69.6 (68.8–70.3)
24–35	46.6 (40.7–52.6)	49.5 (45.3–53.7)	61.1 (60.34-62.0)	51.5 (45-57.9)	59.1 (53.9–64.1)	52.5 (46.9–58.1)	60.5 (59.7–61.3)
36–47	43.1 (38.2–48)	45.2 (40.6–49.9)	51.1 (50.3–51.9)	51.4 (45.9–57)	50.1 (45.7–54.4)	41.2 (35.7–46.8)	50.8 (50-51.5)
48–59	36.8 (31.8–42.2)	40.1 (35.3–44.9)	44.0 (43.2–44.8)	40.3 (45.9–57)	42.8 (37.9–47.8)	36.9 (31.2–42.3)	43.7 (42.9–44.5)
**Recent fever**							
No	47.5 (44.1–50.9)	53** (50.2–55.8)	57.1*** (56.6–57.3)	48.9 (45.9–52)	58* (55.4–60.5)	50.4 (47.2–53.7)	56.8*** (56.4–57.2)
Yes	56.66 (52.6–60.6)	61.1 (57.3–64.8)	60.5 (59.5–61.4)	56.6 (48.6–64.2)	63.6 (58.3–68.5)	54.4 (48.7–60)	60.3 (59.4–61.1)
**Recent diarrhea**							
No	51.1** (48.3–53.8)	54** (51.5–56.4)	56.9*** (56.4–57.3)	50.6 (47.5–53.8)	58.9* (56.3–61.4)	51.5 (48.6–54.4)	56.7*** (56.2–57.1)
Yes	49.8 (38.3–61.3)	65.3 (58.8–71.3)	63.9 (62.7–65.1)	52.9 (37.3–68)	59.5 (52.4–66.2)	48.9 (37.5–60.5)	63.5 (62.4–64.7)
**Children stunted**							
Not stunted	48.8* (45.6–52.0)	52.6** (49.9–55.4)	53.9*** (53.4–54.5)	50.7 (47.3–54.1)	58.8 (56.1–61.5)	49.1* (45.3–52.8)	53.8*** (53.3–54.3)
Stunted	53.3 (48.9–57.6)	60.6 (56.9–64.1)	63.1 (62.5–63.7)	50 (41.5–58.5)	59.1 (54.9–63.2)	55.4 (50.7–60.1)	62.7 (62.2–63.3)
**Children underweight**							
Not underweight	51.4 (48.1–54.6)	53.52** (51-55.9)	54.7*** (54.2–55.2)	50.6 (47.3–54)	58.1 (55.3–60.7)	48.2** (44.7–51.7)	54.6*** (54.1–55.1)
Underweight	49.5 (45.1–53.9)	60.7 (56.3–65.1)	62.6 (61.9–63.2)	53.1 (43.8–62.1)	62.5 (57.6–67.1)	59.5 (54.6–64.3)	62.3 (61.7–62.9)
**Children wasted**							
Not wasted	50.9 (48-53.7)	54.3** (52-56.7)	56.7*** (56.2–57.2)	50.5 (47.2–53.8)	58.5 (56.1–61)	49.6*** (46.6–52.7)	56.5*** (56.1–56.9)
Wasted	49.6 (43.5–55.8)	64.6 (57.6–71.1)	60.7 (59.8–61.5)	51.6 (40.6–62.4)	63.7 (55.1–71.5)	68.1 (60-75.1)	60.6 (59.7–61.5)
**Mothers and household-level factors**							
**Mothers age (years)**							
15–24	54.2* (50.2–58.1)	62.1** (57.9–66)	61.37*** (60.6-62.05)	54.1 (46-62.1)	67.1** (62.1–71.6)	55* (50.8–59.1)	61.1*** (60.5–61.8)
25–34	47.8 (44.1–51.5)	53 (49.8–56.1)	55.9 (55.3–56.4)	50.9 (46.9–54.8)	57.4 (54.1–60.6)	49.9 (46.1–53.6)	55.6 (55.1–56.1)
35–49	49.1 (41.3–56.8)	53.4 (48.5–58.3)	54.6 (53.5–55.8)	48.8 (42.1–55.6)	57.3 (53.2–61.4)	44.8 (35.9–54.1)	54.4 (53.4–55.5)
**Mothers education level**							
No education	52.1 (45.9–58.1)	55.3* (49.1–61.3)	64.4*** (63.7–65)	38.6 (18.9–62.8)	53.6* (48-59.2)	56.3* (51.5–60.9)	64*** (63.3–64.6)
Primary	52.3 (47.9–56.5)	58 (55.1–60.8)	59.6 (58.6–60.5)	44.9 (39.1–50.9)	60.9 (57.2–64.4)	53.2 (47.1–59.2)	59.1 (58.2–59.9)
Secondary or above	49.8 (46.2–53.4)	51.1 (47.2–55)	53.7 (53.1–54.3)	52.4 (48.9–55.9)	58.6 (54.8–62.2)	47.1 (42.9–51.4)	53.6 (53.1–54.1)
**Wealth quintile**							
Poorest	55.8** (50.2–61.1)	63.1*** (58.4–67.5)	63.5*** (62.8–64.1)	45.2** (40.9–49.5)	59.8* (55.4–64)	48.9** (43.3–54.6)	63.1*** (62.4–63.7)
Poor	57.8 (52.2–63.2)	60.6 (55.7–65.3)	58.9 (58.1–59.6)	48.1 (42.9–53.2)	59.5 (54.8–64)	47.8 (41.8–53.9)	58.7 (58-59.4)
Middle	52.8 (47.3–58.4)	54 (48.8–59.1)	58.1 (57.2–58.9)	45.8 (39.9–49.8)	59.8 (54.2–65.3)	60.1 (54.4–65.5)	57.8 (57.1–58.6)
Richer	44.1 (38.6–49.6)	53.9 (48.8–58.9)	53.1 (52.1–54.2)	55.1 (44.2–65.4)	53.9 (48.3–59.4)	56.3 (49.8–62.6)	53.1 (52.1–54.1)
Richest	43 (37.3–48.9)	43 (38.4–47.7)	51.3 (50.1–52.6)	78 (61-88.9)	61.7 (55.4–67.7)	39.2 (32.1–46.7)	51.2 (50-52.4)
**Maternal anemia**							
Not anemic	43.8*** (40.5–47.1)	49.2*** (46.4–52.1)	49.5*** (48.9–50.1)	43.2** (38.9–47.6)	53*** (49.8–56.1)	43.1*** (39.1–46.6)	49.3*** (48.8–49.9)
Anemic	60.5 (56.9–64.1)	63.1 (59.7–66.3)	63.7 (63.2–64.3)	55.4 (51.2–59.6)	67 (63.6–70.2)	61.8 (57.7–65.7)	63.6 (63.1–64.2)
**Community-level factors**							
**Place of residence**							
Urban	45.8* (41.2–50.5)	42.8*** (38.3–47.5)	54.8*** (53.8–55.8)	65.9** (56.1–74.5)	60.6 (55,8-65.3)	48.1* (44.2–51.9)	54.6*** (53.7–55.6)
Rural	52.5 (49.4–55.6)	57.5 (54.9–60)	58.6 (58.2–59.1)	45.5 (42.9–48.2)	58.5 (55.7–61.2)	55.2 (50.8–59.6)	58.4 (58-58.8)
**Community maternal anemia** ^**a**^							
Low	44.2*** (40.7–47.8)	49.7*** (46.4–52.9)	50.4*** (49.8–51.1)	44.6** (40.2–49.1)	54.5** (50.9–58.1)	42.6*** (38.8–46.5)	50.3*** (49.7–50.9)
High	57.3 (53.7–60.9)	60.6 (57.3–63.7)	63.7 (63.1–64.3)	56.9 (52.1–61.5)	63 (59.8–66.1)	60.2 (56.2–64.1)	63.5 (63-64.1)
**Community parity** ^**b**^							
Low	48.6** (45.5–51.6)	54.1 (51.2–57.1)	55.4*** (54.8–55.9)	52.6 (48.5–56.6)	58.4* (55.5–61.2)	48.6** (44.8–52.4)	55.3*** (54.8–55.8)
High	55.8 (50.9–60.6)	57.7 (53.9–61.4)	61.8 (61.2–62.5)	46.5 (41.5–52.1)	60.2 (55.5–64.7)	55.5 (51–60)	61.5 (60.9–62.1)
**Community wealth** ^**c**^							
Low	47.1** (43.5–50.7)	49.4*** (46.2–52.5)	54.8*** (54.2–55.5)	54.9** (50.1–59.9)	58.2* (54.8–61.4)	53.9** (50.2–57.6)	54.8*** (54.1–55.4)
High	54.8 (51.1–58.6)	62.1 (58.8–65.1)	60.3 (59.8–60.8)	44.8 (41-48.72)	59.7 (56.1–63.3)	47.3 (42.6–52.1)	60.1 (59.6–60.6)
**Community female education** ^**d**^							
Low	53.3 (49.3–57.3)	57.5* (54.6–60.4)	62.7*** (62.2–63.3)	46.6* (41.3–52)	59.5* (56.2–62.4)	56.5** (52.4–60.6)	62.3*** (61.8–62.8)
High	49.1 (45.6–52.6)	51.9 (47.9–55.9)	52.9 (52.3–53.3)	53.5 (49.5–57.4)	58.3 (54.6–61.8)	45.9 (41.9–50)	52.9 (52.3–53.5)
**Community safe water access** ^**e**^							
Low	53.6 (45.9–61.3)	-	57.5 (57.1–58.1)	58.3** (52.1–64.2)	61.5 (57.4–65.4)	53.3** (49.8–56.9)	57.6 (56.8–58.3)
High	50.7 (47.9–53.5)	-	57.4 (56.5–57.8)	45.1 (42.1–48.2)	57.3 (54.2–60.3)	46.3 (41.2–51.6)	57.4 (56.9–57.9)
**Community toilet facility** ^**f**^							
Low	51.1 (47.5–54.6)	59.8** (56.6–63.2)	60.6*** (60.1–61.1)	52.2 (39.1–65.1)	59.5 (56.2–62.7)	56.8** (51.5–61.9)	60.6*** (60.1–61.1)
High	50.9 (47-54.9)	50.5 (47.3–53.7)	54.4 (53.7–55.1)	50.7 (47.4–54)	58.3 (54.6–61.9)	48.3 (44.6–52)	54.3 (53.7–54.9)

Frequency (n) are unweighted; percentage (%) are weighted, SSEA: South and Southeast Asia; *p < 0.05, **p < 0.01, ***p < 0.001; ^a^ mean percent of women with Hb levels less than 12 g/dL; ^b^mean percent of women with fertility rate of 5 children and above; ^c^mean percent of households wealth quintiles categorized richer and richest and above; ^d^mean percent of women with primary education level and above; ^e^mean percent of households with access to the improved water source; ^f^mean percent of household with access to an improved toilet facility

**Table 3:** Multivariable multilevel logistic regression (MMLR) analysis of individual and community level factor associated with childhood anemia (n = 167,017)

Individual-and community-level characteristics	Bangladesh	Cambodia	India	Maldives	Myanmar	Nepal
	aOR (95% CI)	aOR (95% CI)	aOR (95% CI)	aOR (95% CI)	aOR (95% CI)	aOR (95% CI)
Fixed effect						
**Individual-level factors**						
**Child-level factors**						
**Sex of child**						
Male	NA	Ref	NA	Ref	NA	NA
Female	NA	0.88 (0.75–1.03)	NA	0.88 (0.70–1.07)	NA	NA
**Child age (months)**						
6–11	Ref	Ref	Ref	Ref	Ref	Ref
12–23	0.77 (0.52–1.15)	0.60 (0.44–0.82)**	1.07 (1.02–1.12)**	0.72 (0.49–1.09)	1.02 (0.72,1.45)	0.95 (0.62–1.47)
24–35	0.27 (0.18–0.40)***	0.24 (0.18–0.33)***	0.65 (0.62–0.68)***	0.67 (0.45–1.05)	0.39 (0.28–0.55)***	0.45 (0.29–0.69)***
36–47	0.24 (0.16–0.36)***	0.19 (0.14–0.26)***	0.41 (0.39–0.43)***	0.69 (0.46–1.04)	0.26 (0.18–0.37)***	0.28 (0.14–0.34)***
48–59	0.18 (0.12–0.28)***	0.14 (0.11–0.21)***	0.30 (0.29–0.32)***	0.48 (0.32–0.75)***	0.21 (0.14–0.29***	0.22 (0.14–0.34)***
**Recent fever**						
No	NA	Ref	Ref	NA	Ref	NA
Yes	NA	1.29 (1.07–1.55)**	1.03 (1.00-1.07)*	NA	1.08 (0.88–1.34)*	NA
**Recent diarrhea**						
No	Ref	Ref	Ref	NA	Ref	NA
Yes	0.81 (0.63–1.24)	1.09 (0.85–1.41)	1.07 (1.02–1.12)**	NA	0.69 (0.53–0.89)	NA
**Children stunted**						
Not stunted	Ref	Ref	Ref	NA	NA	Ref
Stunted	1.33 (1.08–1.63)**	1.42 (1.17–1.73)***	1.29 (1.25–1.33)***	NA	NA	1.27 (0.97–1.67)*
**Children underweight**						
Not underweight	NA	Ref	Ref	NA	NA	Ref
Underweight	NA	1.18 (0.93–1.49)	1.22 (1.18–1.26)***	NA	NA	1.18 (0.87–1.59)
**Children wasted**						
Not wasted	NA	Ref	Ref	NA	NA	Ref
Wasted	NA	1.15 (0.85–1.54)	1.05 (1.02–1.09)**	NA	NA	1.27 (0.83–1.90)
**Mothers and household-level factors**						
**Mothers age (years)**						
15–24	Ref	Ref	Ref	NA	Ref	Ref
25–34	0.84 (0.67–1.03)	0.89 (0.73–1.08)	0.94 (0.91–0.96)***	NA	0.79 (0.62–1.01)	0.98 (0.77–1.24)
35–49	0.91 (0.65–1.30)	0.99 (0.77–1.29)	0.84 (0.81–0.88)***	NA	0.78 (0.59–0.99)*	0.77 (0.51–1.15)
**Mothers education level**						
No education	NA	Ref	Ref	NA	Ref	Ref
Primary	NA	0.99 (0.78–1.28)	0.84 (0.81–0.88)***	NA	1.19 (0.92,1.53)	0.75 (0.54–1.04)
Secondary or above	NA	0.79 (0.59–1.05)	0.76 (0.73–0.78)***	NA	1.02 (0.76–1.38)	0.68 (0.50–0.93)*
**Wealth quintile**						
Poorest	Ref	Ref	Ref	Ref	Ref	Ref
Poor	0.85 (0.63–1.15)	0.99 (0.77–1.26)	0.91 (0.87–0.94)***	1.06 (0.81–1.38)	0.91 (0.72–1.15)	0.76 (0.55–1.01)
Middle	0.75 (0.53–1.06)	0.81 (0.61–1.09)	0.89 (0.85–0.93)***	0.89 (0.66–1.18)	0.87 (0.66–1.16)	1.05 (0.71–1.59)
Richer	0.53 (0.37–0.76)***	0.93 (0.68–1.28)	0.85 (0.81–0.89)***	1.16 (0.67–1.70)	0.77 (0.57–1.05)	0.91 (0.58–1.42)
Richest	0.52 (0.36–0.76)**	0.60 (0.42–0.86)**	0.87 (0.82–0.92)***	2.60 (0.96–7.07)	0.90 (0.62–1.30)	0.55 (0.32–0.94)*
**Maternal anemia**						
Not anemic	Ref	Ref	Ref	Ref	Ref	Ref
Anemic	1.66 (1.33–2.07)***	1.56 (1.32–1.84)***	1.62 (1.59–1.67)***	1.44 (1.16–1.78)**	1.59 (1.34–1.91)***	1.71 (1.36–2.15)***
**Community-level factors**						
**Place of residence**						
Urban	Ref	Ref	Ref	Ref	NA	Ref
Rural	0.97 (0.76–1.24)	1.01 (0.79–1.29)	0.93 (0.88–0.96)***	0.86 (0.45–1.61)	NA	1.08 (0.84–1.39)
**Community maternal anemia** ^**a**^						
Low	Ref	Ref	Ref	Ref	Ref	Ref
High	1.21 (0.97–1.51)*	1.31 (1.09–1.57)**	1.72 (1.66–1.78)***	1.35 (1.08–1.69)**	1.33 (1.08–1.64)**	1.72 (1.34–2.23)***
**Community parity** ^**b**^						
Low	Ref	NA	Ref	NA	Ref	Ref
High	1.04 (0.84–1.30)	NA	0.94 (0.91–0.98)	NA	0.89 (0.72–1.11)	0.97 (0.74–1.27)
**Community wealth** ^**c**^						
Low	Ref	Ref	Ref	Ref	Ref	Ref
High	1.02 (0.78–1.33)	1.13 (0.86–1.48)	0.80 (0.76–0.84)***	0.90 (0.70–1.15)	1.04 (0.81–1.35)	0.78 (0.55–1.10)*
**Community female education** ^**d**^						
Low	NA	Ref	Ref	Ref	Ref	Ref
High	NA	0.98 (0.75–1.21)	0.75 (0.73–0.80)***	0.97 (0.78–1.49)	0.96 (0.81–1.35)	1.02 (0.75–1.39)
**Community safe water access** ^**e**^						
Low	NA	NA	NA	Ref	NA	Ref
High	NA	NA	NA	1.07(0.83–1.38)		1.01(0.77–1.31)
**Community toilet facility** ^**f**^						
Low	NA	Ref	Ref	NA	NA	Ref
High	NA	0.99 (0.78–1.24)	1.16 (1.11–1.21)***	NA		1.05 (0.78–1.42)
**Random effect**						
Community-level variance (SE)	0.097 (0.089)	0.281 (0.071)***	0.929 (0.019)***	0.143 (0.074)**	0.306 (0.072)***	0.355 (0.110)***
ICC (%)	1	4.3	18.2	2.3	8.2	4.4
PCV (%)	71	44	21	47	4	56
**Model fit statistics**						
AIC	2426.6	4053.4	191292.0	2313.6	3713.4	2193.5
BIC	2532.1	4217.7	191550.7	2406.1	3839.1	2330

*p < 0.05, **p < 0.01, ***p < 0.001; aOR = adjusted odds ratio; CI: confidence interval; Ref: reference category; SE: standard error; ICC: intra-class correlation; PCV: percentage change in variation; AIC: Akaike information criterion, BIC: Bayesian information criterion; ^a^mean percent of women with Hb levels less than 12 g/dL; ^b^mean percent of women with fertility rate of 5 children and above; ^c^mean percent of households wealth quintiles categorized richer and richest and above; ^d^mean percent of women with primary education level and above; ^e^mean percent of households with access to improved water source; ^f^ mean percent of household with access to improved toilet facility; NA: Not applicable

### Findings from bivariate analysis

At the individual level, the prevalence of anemia was significantly higher among males (57.7% in Bangladesh, 57.5% in Cambodia, and 54.4% in the Maldives), children with a recent history of fever (56.6% in Bangladesh, 61.1% in Cambodia, 60.5% in India, 56.6% in Maldives, 63.62% in Myanmar, and 60.3% in Nepal), stunting (53.3% in Bangladesh, 60.6% in Cambodia, 63.1% in India, 59.1% in Myanmar, and 55.4% in Nepal), wasting (64.6% in Cambodia, 60.7% in India, 51.6% in Maldives, 63.7% in Myanmar, and 68% in Nepal), and underweight (51.4% in Bangladesh, 64.6% in Cambodia, 60.7% in India, 62.6% in Myanmar, 62.5% in Maldives, and 59.5% in Nepal). Similarly, childhood anemia was higher among those children whose mothers with anemia were (60.5% in Bangladesh, 63.1% in Cambodia, 63.7% in India, 55.4% in Maldives, 67% in Myanmar, and 61.8% in Nepal) (Table 2).

At the community level, the prevalence of childhood anemia was higher among the resident from rural areas (52.5% in Bangladesh, 57.5% in Cambodia, 58.6% in India, and 58.6% in Nepal), communities with a higher percentage of the mother with anemia in Bangladesh (57.3%), Cambodia (60.6%), India (63.7%), Maldives (56.9%), Myanmar (63%), and Nepal (60.2%). The higher percentage of anemia prevalence was among mothers with high parity (55.8% in Bangladesh, 57.7% in Cambodia, 61.8% in India, 60.2% in Myanmar, and 55.5% in Nepal), and mothers coming from a low prevalence of community education (53.3% in Bangladesh, 57.5% in Cambodia, 62.7% in India, 59.5% in Myanmar, and 56.5% in Nepal). Likewise, the prevalence of childhood anemia was higher in communities with lesser access to safe water (53.6% in Bangladesh, 57.5% in India, 58.3% in Maldives, 61.5% in Myanmar, and 53.3% in Nepal), with low community toilet facility (51.1% in Bangladesh, 59.8% in Cambodia, 60.6% in India, 52.2% in Maldives, 59.5% in Myanmar, and 56.8% in Nepal) (Table 2).

### Multivariable analysis

Several factors were significantly associated with childhood anemia. In individual-level factors, childhood anemia was significantly associated with mothers with anemia compared to non-anemic mothers (Bangladesh: aOR = 1.66, Cambodia: aOR = 1.56, India: aOR = 1.62, Maldives: aOR = 1.44, Myanmar: aOR = 1.59, and Nepal: aOR = 1.71), and children with a history of fever in the last two weeks compared to no history of fever (Cambodia: aOR = 1.29, India: aOR = 1.03, Myanmar: aOR = 1.08). Stunted children were more likely to have higher childhood anemia compared to those who were not (Bangladesh: aOR = 1.33, Cambodia: aOR = 1.42, India: aOR = 1.29, and Nepal: aOR = 1.27). In contrast, several factors were significantly associated with lower odds of childhood anemia: children of 48–59 months of age compared to younger children in all countries (Bangladesh: aOR = 0.18, Cambodia: aOR = 0.14, India: aOR = 0.30, Maldives: aOR = 0.48, Myanmar: aOR = 0.21, and Nepal: aOR = 0.22) (Table 3).

In community-level factors, children with mothers in communities with a high percentage of community maternal anemia had higher odds of childhood anemia compared to mothers in the communities with a low percentage of community maternal anemia in all countries (Bangladesh: aOR = 1.21, Cambodia: aOR = 1.31, India: aOR = 1.72, Maldives: aOR = 1.35, Myanmar: aOR = 1.33, and Nepal: aOR = 1.72) (Table 3).

After including both individual and community-level variables in model IV, estimated intra-class correlation (ICC) showed that about 1% in Bangladesh, 4% in Cambodia, 18% in India, 2% in Maldives, 8% in Myanmar, and 4% in Nepal of the community level variability in childhood anemia was attributable to the difference between communities. On the other hand, the percentage change variances (PCVs) indicated that Bangladesh (59%), Cambodia (44%), India (21%), Maldives (47%), Myanmar (4%), and Nepal (55%) of the variation in childhood anemia across communities was explained by both individual and community-level factors (Table 3).

## Discussion

Planning and implementation of anemia eradication programs entail identifying factors affecting anemia. This study provides country-level prevalence associated with individual and community-level factors related to childhood anemia in six selected South and South Asian countries. The overall weighted prevalence of anemia among children in this study was 57.3% (56.9–57.7%) which is similar to the prevalence of anemia among children under five in South Asia (58% in 2011 [21] and 55.1% in 2016 [12]). According to WHO guidelines, the prevalence of anemia above 40% is a serious public health problem [11, 47].

This study revealed that children born to mothers with anemia were more likely to develop anemia compared to those from non-anemic mothers in all six countries which is consistent with findings from the previous studies [5, 48, 49]. This could be due to multiple reasons. For instance, maternal anemia during pregnancy contributes to low birth weight and preterm birth, both of which increase the likelihood of childhood anemia [5]. Mothers and their children share a common household environment, socioeconomic, and food habits [7]. Mothers who belonged to poor households bear problems in purchasing and providing an iron-rich diet and other micro-nutrient-rich food for themselves and their children which can increase the predilection to develop anemia [5, 48]. Severe maternal anemia may also reduce iron content in breast milk, inevitably inadequate to meet daily requirements of iron which can result in childhood anemia [7].

The prevalence of anemia was lower among older children than among those at a young age. The result shows that as age increases the risk of anemia among children decreases. Younger children between the ages of 6 to 24 months are considered to be at the growth spurt, thus their nutritional requirement is high [23]. However, the region with a higher incidence of maternal micronutrient deficits which can lead to a low concentration of iron in breast milk fails to meet the daily requirements of iron for the children [50]. Another possible explanation for the low prevalence of anemia among children above the age of 2 years may be due to their dietary habit that entails consuming a varied diet including iron-rich food which can eventually improve their hemoglobin level [6]. In addition to these, various public health interventions, such as iron supplementation, deworming, malaria prevention during pregnancy, and promotion of home food fortification (provision of multiple micronutrient powders (MNPs)), were additional contributing factors in reducing the prevalence of anemia and micronutrient deficiencies in children under five [51]. Various studies have shown consistent reports of decreasing anemia with the age of the children [6, 52].

In Cambodia, India, and Myanmar, this study found that a history of fever was positively associated with anemia which is consistent with the previous studies conducted in Myanmar, Benin, Mali, and Malawi [40, 50, 53]. Fever among under-five children in Cambodia, India, and Myanmar was a common symptom of acute and chronic inflammatory disease [27, 31, 50]. India shares a major burden of malaria in South Asia [54]. A similar burden is echoed in Cambodia and Myanmar in South East Asia [55]. Association between childhood anemia and inflammatory infections that included fever, cough, and worm infestation has been well established [54]. The possible pathway of this association is due to inappropriate iron losses during the time of infections [56]. One previous study reported that iron-deficiency anemia may depend on inadequate iron-rich food intake [17], increased iron use in the body, and iron depletion due to parasitic infection [54]. Also, previous studies have reported that malaria increases erythrocyte destruction with a simultaneous failure of the bone marrow thus jeopardizing the ability to compensate for the losses [57].

The likelihood of children being anemic with a history of diarrhea was significantly higher in India, which is consistent with the previous studies conducted in western Rajasthan, India [6, 58]. Diarrheal illness is accompanied by an increased loss of iron and decreased ability of the gastrointestinal tract to absorb nutrients needed to maintain normal hemoglobin status [59]. Stunted children were more likely to be anemic than their counterparts in Bangladesh, Cambodia, India, and Nepal, which is in agreement with the findings from Bangladesh, Brazil [60] Burma [50], and Ethiopia [61]. The plausible reason could be that undernourished children are often anemic, low hemoglobin level has a negative effect on the linear growth during all stages of growth (infancy, childhood, and adolescence), and the coexistence of other micronutrient deficiencies and stunting condition may increase the development of anemia by a synergistic association [62, 63]. In addition, stunting could arise due to chronic food deficit and the adverse impact of micronutrient deficiencies, which are associated with childhood anemia [40]. Inadequate nutrient intake may also impair immunity which in turn leads to a low level of hemoglobin concentration and increased vulnerability to diseases; thus perpetuating a vicious cycle of low nutrient diet and diseases [7].

In this study, children with acute malnutrition were found more likely to be anemic than well-nourished children. Children who were underweight and wasted were more likely to be anemic than their counterparts in India which is supported by the studies conducted in Ethiopia [63] and Brazil [64]. Since anemia and malnutrition often share common causes, it is expected that multiple nutritional problems could co-exist in the same individuals [65]. These factors are aggravated by poverty and food insecurity [66]. Low intake of iron-rich foods and diminished nutrient absorption caused by changes in the gastrointestinal epithelium in malnourished children contributes to the development of anemia [63]. The possible mechanisms are maturation block in the erythroblast leading to a poor reticulocyte response which can give rise to a mild decrease in the number of erythropoietin-sensitive precursor cells, and increased hemolysis [67]. Although in this study, it was not possible to determine whether stunting existed before the anemic condition, the consistent association between these two nutritional conditions emphasizes the need for long-term efforts to improve children’s nutritional status.

The findings of this study suggest that infants born to elder mothers (age: 35–49 years) in India were less likely to develop anemia which echoes with the previous studies [68]. This could be explained by the fact that being an older mother means being mature enough to pay attention to the health and nutrient demands of their children [68]. The increased risk of anemia in younger mothers may signify they are less prepared to perform the duties of motherhood and meet the need of their children. This may reflect a lack of financial resources, lack of knowledge about anemia and child care, and lack of adequate guidance [69].

Also, this study revealed infants born from mothers who had attended secondary or higher levels of education in India and Nepal were less likely to have anemic children compared to women with no formal education which is consistent with results from the previous studies [22, 61]. A well-informed mother is expected to choose a varied diet rich in micronutrients for her child, have more nutritional knowledge about child care, and provide a healthy environment for her children [6]. Therefore, providing nutrition education during pregnancy could bridge the gap in nutritional knowledge among the mothers in this region [17].

The findings of this study suggest that infants born to mothers from poor households in Bangladesh, Cambodia, India, and Nepal were more likely to develop anemia which is also supported by the results from previous studies [48, 70]. A probable explanation may be that children from poor households are less likely to consume a diverse diet especially iron-rich food from animal sources [71]. Also, these households are less likely to afford health services during illness, particularly relevant for many nations in south and southeast Asia where universal health coverage is lacking [72].

Community-level factors showed that communities with a higher percentage of maternal anemia were linked with a higher probability of childhood anemia in all six countries which is consistent with the study from western African countries [5]. As a mother and their children share a common socioeconomic status and home environment, their dietary pattern and quality of life are likely to be affected [73]. Low level of essential micronutrients such as iron, and Vitamin B12 in diet invariably affects mothers’ breast milk which can reduce the hemoglobin level of a breastfeeding child. Additionally, mothers and their children may share similar exposure to infections such as helminthiasis, malaria, and other infectious diseases that may interfere with their red blood cell production and iron stores [74].

The study revealed that residents in rural areas in India are less likely to be anemic than those residing in urban areas. This result is in contrast to the finding from the study conducted in the northeastern states of India [72]. A possible explanation for the significantly higher prevalence of anemia among residents in urban areas may be due to the reason that a high number of slum settlements in the urban areas of India, in fact, are poor and are devoid of basic facilities [54]. Children living in squatter settlements are more likely from extremely poor households with poor hygiene, lack of safe drinking water, and mothers with a lower level of education [75]. A study conducted in New Delhi, India demonstrated that iron and vitamin B12 deficiency are critical contributors to childhood anemia among families in urban slums [54].

Based on this study, promoting community female education can lower the likelihood of childhood anemia which echoes the study from Malawi [5]. This could be explained by the fact that higher community education provides a context where women are more likely to obtain knowledge and material resources that can benefit a child’s health [40]. Our findings also show that community with poor household has higher odds of childhood anemia in India and Nepal. Poor households have to live in extreme social and economic deprivation, hence it is difficult for families to provide a diverse diet, adequate care, and health services to their children [5].

In this study, estimated intra-class correlation (ICC) shows that about 1% in Bangladesh, 4% in Cambodia, 18% in India, 2% in Maldives, 8% Myanmar, and 4% in Nepal of the community level variability in childhood anemia was attributable to the difference between communities. These findings are in line with a previous study on a multilevel model that also demonstrated consistent small ICCs [5]. India has a higher (18%) community-level variance than other countries. The potential explanation could be that the sample size was higher and covered a wider area within the 36 states/union territories, 640 districts, and 26,986 communities [32]. This finding is consistent with previous multilevel analysis from India [6]. However, various studies suggested that an ICCs at or above is suggestive of a potential higher level effect (e.g. group-level variance) and worth examining in multilevel study design [40].

In SSEA, the existing public health interventions program has been implemented to combat anemia, which includes child feeding practices, intake of micronutrients by supplementation or fortification with iron, and point-of-use fortification of complementary foods with multiple micronutrients [76], and delayed cord clamping [77]. Despite these efforts, childhood anemia is high in this region. The potential public health intervention should focus on (1) Encouraging the consumption of a diverse and sustainable healthy diet; (2) Promoting the point-of-use fortification of complementary foods with multiple micronutrients for infants and preschoolers; (3) Controlling inflammation by preventing and treating helminthic, HIV, and malaria infections; (4) Providing nutrition education and counseling of pregnant women on optimal dietary practices, and (5) Increasing the public’s awareness of anemia-prevention programs through different approaches.

This study has some limitations. First, the cross-sectional design of the study limits the ability to establish a robust causal pathway. Second, this study relied on hemoglobin as the measure of anemia; further studies should consider other red blood cell indices [40]. Third, this study included self-reported data for the occurrence of diarrhea and fever; therefore, we could not completely rule out the recall bias and social desirability bias. Fourth, since this study is based on secondary data analysis, we are unable to adjust all confounding factors such as dietary intake, worm infestations, and others. Fifth, this study has used data from six different countries with a five-year time difference, which may have varying educational and economic status over time. Lastly, the HemoCue device has been widely used in field settings where resources are limited since it is portable, simple to use, and reasonably priced, However, air bubbles, too much blood on the cuvettes’ back sides, overfilling the cuvettes, and inadequate mixing of the samples can all provide inaccurate results [36]. Additionally, there are variability in Hb measurements dependent on Hemocue models which inevitably may have affected the prevalence of anemia presented in this study [13, 78]. Despite these limitations, the study included the surveys that are nationally representative samples, thus, findings suggest the generalizability of the data to resource-limited settings in South and Southeast Asia. It uses methodologies in the assessment and classification of anemia across time, the estimates of anemia prevalence are more likely to represent a population-based estimate.

## Conclusion

This study has highlighted a high prevalence of childhood anemia in six countries. Despite various health and nutrition interventions for anemia reduction among children, anemia continues to be an intractable public health problem in this region. The increased likelihood of childhood anemia was associated with both individual-and community-level factors. Our study results indicate that individual factors including maternal anemia, younger child age, presence of infections (diarrhea and fever), malnourished status, young mother, maternal illiteracy, and family poverty were associated with childhood anemia. At the community level, the higher percentage of community maternal anemia, low community female education, and the low percentage of community households were associated with childhood anemia. Factors identified in this study can inform the priority areas and tailor the anemia control programs at the national and regional levels. For instance, public health policy and interventions aimed at reducing childhood anemia may benefit by focusing on (1) strengthening ongoing nutrition-specific and nutrition-sensitive intervention; (2) strengthening maternal anemia with emphasis on addressing iron and other micronutrient deficiencies, and infections; (3) improving infant and young child feeding practices; (4) promoting maternal literacy and livelihood alongside behavior change communication for healthy dietary habit; and (5) increasing the role of public health professionals for social behavior change communications to reduce the childhood anemia. Community-level factors imply the need to focus on contextual factors and promote community female education and community wealth index to reduce childhood anemia.

## Electronic supplementary material

Below is the link to the electronic supplementary material.

Supplementary Material 1

Supplementary Material 2

Supplementary Material 3

## Data Availability

Dataset used in this study is publicly available and can be downloaded upon the formal request from the DHS website (URL:https://www.dhsprogram.com/data/available-datasets.cfm).

## List of Abbreviations

ANC: Antenatal checkup
aOR: Adjusted odds ratio
BMI: Body mass index
CI: Confidence interval
cOR: Crude odds ratio
DHSs: Demographic and Health Surveys
EAs: Enumeration areas
LMICs: Low and middle-income countries
PSU: Primary sampling unit
SDG: Sustainable development goal
SSA: Sub-Saharan Africa
SSEA: South and Southeast Asia
VIF: Variation inflation factor
WHO: World Health Organization
WRA: Women of reproductive age
