# Supplementary material for: Factors associated with anemia among children in South and Southeast Asia: a multilevel analysis
Source: BMC Public Health. 2023 Feb 15;23:343. doi: 10.1186/s12889-023-15265-y (PMC9933407; doi:10.1186/s12889-023-15265-y)
Supplement: Supplementary file 1 — Supplementary Material 1 [file 12889_2023_15265_MOESM1_ESM.docx]

**S1 Table: Sample size selection process**

| Country and DHS year | | Bangladesh 2011 | India  2015-16 | Cambodia  2014 | Maldives  2016-17 | Myanmar  2015-16 | Nepal  2016 |
| --- | --- | --- | --- | --- | --- | --- | --- |
| Total number of interviewed households | | 17,141 | 601,509 | 15,825 | 6,050 | 12,500 | 11,040 |
| Total number children from age 6-59 months | | N=7927 | N=227,290 | N=6450 | N=2828 | N=4321 | N=4583 |
| **Excluded children** | **Number of children without one mother-child pairs** | **1126** | **52,959** | **981** | **338** | **740** | **823** |
|  | **Mother-child pairs with flagged cases of child anemia (inconsistent response is reported as flagged cases in DHS dataset)** | **256** | **-** | **275** | **-** | **-** | **-** |
|  | **Missing information of child Hb level, anthropometry and covariates** | **4608** | **18,938** | **1884** | **794** | **647** | **2013** |
| Final sample size | | 1937 | 155,393 | 3310 | 1696 | 2934 | 1747 |

Note: Exclusion criteria with numbers have been bold
