# Supplementary material for: Factors associated with anemia among children in South and Southeast Asia: a multilevel analysis
Source: BMC Public Health. 2023 Feb 15;23:343. doi: 10.1186/s12889-023-15265-y (PMC9933407; doi:10.1186/s12889-023-15265-y)
Supplement: Supplementary file 2 — Supplementary Material 2 [file 12889_2023_15265_MOESM2_ESM.docx]

**S2 Table: Multivariable multilevel logistic regression (MMLR) analysis of individual and community level factor associated with childhood anemia**

**Bangladesh**

| **Individual-and community-level characteristics** | **Model 1**  **Empty model** | **Model 2**  **Individual-level variable** | **Model 3**  **Community level variable** | **Model 4**  **Individual- and community-level variable** |
| --- | --- | --- | --- | --- |
| **Individual-level factors** |  |  |  |  |
| **Sex of child** |  |  |  |  |
| Male |  | Ref |  | NA |
| Female |  | 0.88 (0.72-1.06) |  | NA |
| **Child age (months)** |  |  |  |  |
| 6-11 |  | Ref |  | Ref |
| 12-23 |  | 0.77 (0.52-1.15) |  | 0.77 (0.52-1.15) |
| 24-35 |  | 0.26 (0.18-0.39)*** |  | 0.27 (0.18-0.40)*** |
| 36-47 |  | 0.23 (0.16-0.35)*** |  | 0.24 (0.16-0.36)*** |
| 48-59 |  | 0.18 (0.12-0.27)*** |  | 0.18 (0.12-0.28)*** |
| **Recent fever** |  |  |  |  |
| No |  | Ref |  | NA |
| Yes |  | 1.17 (0.95-1.43) |  | NA |
| **Recent diarrhea** |  |  |  |  |
| No |  | Ref |  | Ref |
| Yes |  | 0.81 (0.53-1.24)* |  | 0.81 (0.63-1.24) |
| **Children stunted** |  |  |  |  |
| Not stunted |  | Ref |  | Ref |
| Stunted |  | 1.41 (1.11-1.81)*** |  | 1.33 (1.08-1.63)** |
| **Children underweight** |  |  |  |  |
| Not underweight |  | Ref |  | NA |
| Underweight |  | 1.18(0.87-1.60) |  | NA |
| **Children wasted** |  |  |  |  |
| Not wasted |  | Ref |  | NA |
| Wasted |  | 1.26(0.84) |  | NA |
| **Mothers age (years)** |  |  |  |  |
| 15-24 |  | Ref |  | Ref |
| 25-34 |  | 0.81 (0.65-1.01)* |  | 0.84 (0.67-1.03) |
| 35-49 |  | 0.89 (0.63-1.26) |  | 0.91 (0.65-1.30) |
| **Mothers education level** |  |  |  |  |
| No education |  | Ref |  | NA |
| Primary |  | 0.95 (0.71-1.29) |  | NA |
| Secondary or above |  | 0.84 (0.61-1.15) |  | NA |
| **Wealth quintile** |  |  |  |  |
| Poorest |  | Ref |  | Ref |
| Poor |  | 0.86 (0.63-1.17) |  | 0.85 (0.63-1.15) |
| Middle |  | 0.81 (0.59-1.12) |  | 0.75 (0.53-1.06) |
| Richer |  | 0.58 (0.42-0.81)*** |  | 0.53 (0.37-0.76)*** |
| Richest |  | 0.59 (0.42-0.82)*** |  | 0.52 (0.36-0.76)** |
| **Maternal anemia** |  |  |  |  |
| Not anemic |  | Ref |  | Ref |
| Anemic |  | 2.07(1.66-2.56)*** |  | 1.66 (1.33-2.07)*** |
| **Community-level factors** |  |  |  |  |
| **Place of residence** |  |  |  |  |
| Urban |  |  | Ref | Ref |
| Rural |  |  | 1.15 (0.93-1.43)* | 0.97 (0.76-1.24) |
| **Community maternal anemia** |  |  |  |  |
| Low |  |  | Ref | Ref |
| High |  |  | 1.54 (1.28-1.85)*** | 1.21 (0.97-1.51)* |
| **Community parity** |  |  |  |  |
| Low |  |  | Ref | Ref |
| High |  |  | 1.04 (0.85-1.27)* | 1.04 (0.84-1.30) |
| **Community wealth** |  |  |  |  |
| Low |  |  | Ref | Ref |
| High |  |  | 1.11 (0.89-1.37)* | 1.02 (0.78-1.33) |
| **Community female education** |  |  |  |  |
| Low |  |  | Ref | NA |
| High |  |  | 0.83 (0.67-1.01) | NA |
| **Community safe water access** |  |  |  |  |
| Low |  |  | Ref | NA |
| High |  |  | 1.30 (0.96-1.77) | NA |
| **Community toilet facility** |  |  |  |  |
| Low |  |  | Ref | NA |
| High |  |  | 0.99 (0.81-1.22) | NA |
| **Random effect** |  |  |  |  |
| Community-level variance (SE) | 0.063(0.08) | 0.024(0.87)** | 0.005(0.08)* | 0.097 (0.089) |
| ICC (%) | 2.98% | 0.7% | 0.15% | 1% |
| PCV (%) | Reference | 62% | 60% | 71% |
| **Model fit statistics** |  |  |  |  |
| AIC | 2886.06 | 2426.85 | 2660.22 | 2426.6 |
| BIC | 2697.19 | 2543.37 | 2710.34 | 2532.1 |

**Cambodia**

| **Individual-and community-level characteristics** | **Model 1**  **Empty model** | **Model 2**  **Individual-level variable** | **Model 3**  **Community level variable** | **Model 4**  **Individual- and community-level variable** |
| --- | --- | --- | --- | --- |
| **Individual-level factors** |  |  |  |  |
| **Sex of child** |  |  |  |  |
| Male |  | Ref |  | Ref |
| Female |  | 0.89 (0.46-1.03)* |  | 0.88 (0.75-1.03) |
| **Child age (months)** |  |  |  |  |
| 6-11 |  | Ref |  | Ref |
| 12-23 |  | 0.60 (0.44-0.82)*** |  | 0.60 (0.44-0.82)** |
| 24-35 |  | 0.24 (0.17-0.33)*** |  | 0.24 (0.18-0.33)*** |
| 36-47 |  | 0.19 (0.13-0.26)*** |  | 0.19 (0.14-0.26)*** |
| 48-59 |  | 0.15 (0.11-21)*** |  | 0.14 (0.11-0.21)*** |
| **Recent fever** |  |  |  |  |
| No |  | Ref |  | Ref |
| Yes |  | 1.31 (1.08-1.56)* |  | 1.29 (1.07-1.55)** |
| **Recent diarrhea** |  |  |  |  |
| No |  | Ref |  | Ref |
| Yes |  | 1.09 (0.84-1.41)* |  | 1.09 (0.85-1.41) |
| **Children stunted** |  |  |  |  |
| Not stunted |  | Ref |  | Ref |
| Stunted |  | 1.42 (1.17-1.73)*** |  | 1.42 (1.17-1.73)*** |
| **Children underweight** |  |  |  |  |
| Not underweight |  | Ref |  | Ref |
| Underweight |  | 1.18 (0.87-1.60)* |  | 1.18 (0.93-1.49) |
| **Children wasted** |  |  |  |  |
| Not wasted |  | Ref |  | Ref |
| Wasted |  | 1.26(0.86-1.55)* |  | 1.15 (0.85-1.54) |
| **Mothers age (years)** |  |  |  |  |
| 15-24 |  | Ref |  | Ref |
| 25-34 |  | 0.89 (0.74-1.26)* |  | 0.89 (0.73-1.08) |
| 35-49 |  | 0.98 (0.77-1.26) |  | 0.99 (0.77-1.29) |
| **Mothers education level** |  |  |  |  |
| No education |  | Ref |  | Ref |
| Primary |  | 0.99 (0.78-1.26) |  | 0.99 (0.78-1.28) |
| Secondary or above |  | 0.77 (0.58-1.02)* |  | 0.79 (0.59-1.05) |
| **Wealth quintile** |  |  |  |  |
| Poorest |  | Ref |  | Ref |
| Poor |  | 0.96 (0.75-1.22) |  | 0.99 (0.77-1.26) |
| Middle |  | 0.73 (0.56-0.95)* |  | 0.81 (0.61-1.09) |
| Richer |  | 0.80 (0.62-1.04) |  | 0.93 (0.68-1.28) |
| Richest |  | 0.50 (0.38-0.65)*** |  | 0.60 (0.42-0.86)** |
| **Maternal anemia** |  |  |  |  |
| Not anemic |  | Ref |  | Ref |
| Anemic |  | 2.07(1.66-2.56)*** |  | 1.56 (1.32-1.84)*** |
| **Community-level factors** |  |  |  |  |
| **Place of residence** |  |  |  |  |
| Urban |  |  | Ref | Ref |
| Rural |  |  | 1.16 (0.93-1.44)* | 1.01 (0.79-1.29) |
| **Community maternal anemia** |  |  |  |  |
| Low |  |  | Ref | Ref |
| High |  |  | 1.52 (1.30-1.78)*** | 1.31 (1.09-1.57)** |
| **Community parity** |  |  |  |  |
| Low |  |  | Ref | NA |
| High |  |  | 0.90 (0.76-1.07)* | NA |
| **Community wealth** |  |  |  |  |
| Low |  |  | Ref | Ref |
| High |  |  | 1.40 (1.16-1.69)*** | 1.13 (0.86-1.48) |
| **Community female education** |  |  |  |  |
| Low |  |  | Ref | Ref |
| High |  |  | 0.83 (0.67-1.03)* | 0.98 (0.75-1.21) |
| **Community safe water access** |  |  |  |  |
| Low |  |  | - | NA |
| High |  |  | - | NA |
| **Community toilet facility** |  |  |  |  |
| Low |  |  | Ref | Ref |
| High |  |  | 1.16 (0.94-1.44)* | 0.99 (0.78-1.24) |
| **Random effect** |  |  |  |  |
| Community-level variance (SE) | 0.281(0.07)*** | 0.172(0.07)** | 0.134(0.06)** | 0.157 (0.067)** |
| ICC (%) | 7.90% | 4.8% | 3.99% | 4.3 |
| PCV (%) | Reference | 39% | 52% | 44 |
| **Model fit statistics** |  |  |  |  |
| AIC | 4542.33 | 4053.54 | 4469.74 | 4053.4 |
| BIC | 4554.54 | 4181.32 | 4518.58 | 4217.7 |

**India**

| **Individual-and community-level characteristics** | **Model 1**  **Empty model** | **Model 2**  **Individual-level variable** | **Model 3**  **Community level variable** | **Model 4**  **Individual- and community-level variable** |
| --- | --- | --- | --- | --- |
| **Individual-level factors** |  |  |  |  |
| **Sex of child** |  |  |  |  |
| Male |  | Ref |  | NA |
| Female |  | 1.03(0.83-1.27) |  | NA |
| **Child age (months)** |  |  |  |  |
| 6-11 |  | Ref |  | Ref |
| 12-23 |  | 0.93(0.61-1.42) |  | 1.07 (1.02-1.12)** |
| 24-35 |  | 0.44(0.29-0.68)*** |  | 0.65 (0.62-0.68)*** |
| 36-47 |  | 0.28(0.18-0.43)*** |  | 0.41 (0.39-0.43)*** |
| 48-59 |  | 0.21(0.14-0.34)*** |  | 0.30 (0.29-0.32)*** |
| **Recent fever** |  |  |  |  |
| No |  | Ref |  | Ref |
| Yes |  | 1.05(0.80-1.36) |  | 1.03 (1.00-1.07)* |
| **Recent diarrhea** |  |  |  |  |
| No |  | Ref |  | Ref |
| Yes |  | 0.97(0.63-1.49) |  | 1.07 (1.02-1.12)** |
| **Children stunted** |  |  |  |  |
| Not stunted |  | Ref |  | Ref |
| Stunted |  | 1.27(0.97-1.65) |  | 1.29 (1.25-1.33)*** |
| **Children underweight** |  |  |  |  |
| Not underweight |  | Ref |  | Ref |
| Underweight |  | 1.24 (1.19-1.28)*** |  | 1.22 (1.18-1.26)*** |
| **Children wasted** |  |  |  |  |
| Not wasted |  | Ref |  | Ref |
| Wasted |  | 1.05 (1.02-1.09)*** |  | 1.05 (1.02-1.09)** |
| **Mothers age (years)** |  |  |  |  |
| 15-24 |  | Ref |  | Ref |
| 25-34 |  | 0.92 (0.90-0.95)*** |  | 0.94 (0.91-0.96)*** |
| 35-49 |  | 0.69 (0.67-0.72)*** |  | 0.84 (0.81-0.88)*** |
| **Mothers education level** |  |  |  |  |
| No education |  | Ref |  | Ref |
| Primary |  | 0.82 (0.79-0.86) |  | 0.84 (0.81-0.88)*** |
| Secondary or above |  | 0.69 (0.67-0.72) |  | 0.76 (0.73-0.78)*** |
| **Wealth quintile** |  |  |  |  |
| Poorest |  | Ref |  | Ref |
| Poor |  | 0.89 (0.87-0.93)*** |  | 0.91 (0.87-0.94)*** |
| Middle |  | 0.89 (0.86-0.93)*** |  | 0.89 (0.85-0.93)*** |
| Richer |  | 0.84 (0.81-0.88)*** |  | 0.85 (0.81-0.89)*** |
| Richest |  | 0.87 (0.83-0.91) |  | 0.87 (0.82-0.92)*** |
| **Maternal anemia** |  |  |  |  |
| Not anemic |  | Ref |  | Ref |
| Anemic |  | 2.07(1.66-2.56)*** |  | 1.62 (1.59-1.67)*** |
| **Community-level factors** |  |  |  |  |
| **Place of residence** |  |  |  |  |
| Urban |  |  | Ref | Ref |
| Rural |  |  | 0.95 (0.91-0.99)* | 0.93 (0.88-0.96)*** |
| **Community maternal anemia** |  |  |  |  |
| Low |  |  | Ref | Ref |
| High |  |  | 2.14 (2.07-2.21)*** | 1.72 (1.66-1.78)*** |
| **Community parity** |  |  |  |  |
| Low |  |  | Ref | Ref |
| High |  |  | 0.96 (0.93-0.99)* | 0.94 (0.91-0.98) |
| **Community wealth** |  |  |  |  |
| Low |  |  | Ref | Ref |
| High |  |  | 0.96 (0.92-0.99)* | 0.80 (0.76-0.84)*** |
| **Community female education** |  |  |  |  |
| Low |  |  | Ref | Ref |
| High |  |  | 0.68 (0.65-0.70)*** | 0.75 (0.73-0.80)*** |
| **Community safe water access** |  |  |  |  |
| Low |  |  | Ref | NA |
| High |  |  | 0.93 (0.90-0.96)*** | NA |
| **Community toilet facility** |  |  |  |  |
| Low |  |  | Ref | Ref |
| High |  |  | 1.16 (1.12-1.21)*** | 1.16 (1.11-1.21)*** |
| **Random effect** |  |  |  |  |
| Community-level variance (SE) | 0.929(0.019)*** | 0.813(0.026)*** | 0.714(0.025)* | 0.731 (0.018)*** |
| ICC (%) | 22% | 19% | 17.69% | 18.2% |
| PCV (%) | Reference | 12% | 23% | 21% |
| **Model fit statistics** |  |  |  |  |
| AIC | 204852.9 | 192606.2 | 201795.7 | 191292 |
| BIC | 204872.8 | 192815.1 | 201885.3 | 191550.7 |

**Maldives**

| **Individual-and community-level characteristics** | **Model 1**  **Empty model** | **Model 2**  **Individual-level variable** | **Model 3**  **Community level variable** | **Model 4**  **Individual- and community-level variable** |
| --- | --- | --- | --- | --- |
| **Individual level** |  |  |  |  |
| **Sex of child** |  |  |  |  |
| Male |  | Ref |  | Ref |
| Female |  | 1.03(0.83-1.27)* |  | 0.88 (0.70-1.07) |
| **Child age (months)** |  |  |  |  |
| 6-11 |  | Ref |  | Ref |
| 12-23 |  | 0.93(0.61-1.42) |  | 0.72 (0.49-1.09) |
| 24-35 |  | 0.44(0.29-0.68)*** |  | 0.67 (0.45-1.05) |
| 36-47 |  | 0.28(0.18-0.43)*** |  | 0.69 (0.46-1.04) |
| 48-59 |  | 0.21(0.14-0.34)*** |  | 0.48 (0.32-0.75)*** |
| **Recent fever** |  |  |  |  |
| No |  | Ref |  | NA |
| Yes |  | 1.05(0.80-1.36) |  | NA |
| **Recent diarrhea** |  |  |  |  |
| No |  | Ref |  | NA |
| Yes |  | 0.97(0.63-1.49) |  | NA |
| **Children stunted** |  |  |  |  |
| Not stunted |  | Ref |  | NA |
| Stunted |  | 1.27(0.97-1.65) |  | NA |
| **Children underweight** |  |  |  |  |
| Not underweight |  | Ref |  | NA |
| Underweight |  | 1.13 (0.78-1.64) |  | NA |
| **Children wasted** |  |  |  |  |
| Not wasted |  | Ref |  | NA |
| Wasted |  | 0.95 (0.63-1.44) |  | NA |
| **Mothers age (years)** |  |  |  |  |
| 15-24 |  | Ref |  | NA |
| 25-34 |  | 0.91 (0.67-1.25) |  | NA |
| 35-49 |  | 0.97 (0.65-1.44) |  | NA |
| **Mothers education level** |  |  |  |  |
| No education |  | Ref |  | NA |
| Primary |  | 1.60 (0.66-3.86) |  | NA |
| Secondary or above |  | 1.58 (0.66-3.76) |  | NA |
| **Wealth quintile** |  |  |  |  |
| Poorest |  | Ref |  | Ref |
| Poor |  | 1.07 (0.82-1.39) |  | 1.06 (0.81-1.38) |
| Middle |  | 0.95 (0.72-1.24) |  | 0.89 (0.66-1.18) |
| Richer |  | 1.26 (0.84-1.88) |  | 1.16 (0.67-1.70) |
| Richest |  | 3.83 (1.65-8.87)*** |  | 2.60 (0.96-7.07) |
| **Maternal anemia** |  |  |  |  |
| Not anemic |  | Ref |  | Ref |
| Anemic |  | 2.07(1.66-2.56)*** |  | 1.44 (1.16-1.78)** |
| **Community-level factors** |  |  |  |  |
| **Place of residence** |  |  |  |  |
| Urban |  |  | Ref | Ref |
| Rural |  |  | 0.55 (0.34-0.91)* | 0.86 (0.45-1.61) |
| **Community maternal anemia** |  |  |  |  |
| Low |  |  | Ref | Ref |
| High |  |  | 1.42 (1.11-1.82)** | 1.35 (1.08-1.69)** |
| **Community parity** |  |  |  |  |
| Low |  |  | Ref | NA |
| High |  |  | 1.03 (0.80-1.32) | NA |
| **Community wealth** |  |  |  |  |
| Low |  |  | Ref | Ref |
| High |  |  | 0.87 (0.69-1.10)* | 0.90 (0.70-1.15) |
| **Community female education** |  |  |  |  |
| Low |  |  | Ref | Ref |
| High |  |  | 1.02 (0.74-1.40)* | 0.97 (0.78-1.49) |
| **Community safe water access** |  |  |  |  |
| Low |  |  | Ref | Ref |
| High |  |  | 1.12 (0.87-1.40)* | 1.07(0.83-1.38) |
| **Community toilet facility** |  |  |  |  |
| Low |  |  | Ref | NA |
| High |  |  | 0.86 (0.50-1.48) | NA |
| **Random effect** |  |  |  |  |
| Community-level variance (SE) | 0.143(0.074)** | 0.112(0.073)* | 0.096(0.066)* | 0.076 (0.066) |
| ICC (%) | 4.23% | 3.30% | 2.84% | 2.3% |
| PCV (%) | Reference | 22% | 33% | 47% |
| **Model fit statistics** |  |  |  |  |
| AIC | 2345.47 | 2245.45 | 2335.76 | 2313.6 |
| BIC | 2356.34 | 2385.88 | 2348.68 | 2406.1 |

**Myanmar**

| **Individual-and community-level characteristics** | **Model 1**  **Empty model** | **Model 2**  **Individual-level variable** | **Model 3**  **Community level variable** | **Model 4**  **Individual- and community-level variable** |
| --- | --- | --- | --- | --- |
| **Individual-level factors** |  |  |  |  |
| **Sex of child** |  |  |  |  |
| Male |  | Ref |  | NA |
| Female |  | 1.03(0.83-1.27) |  | NA |
| **Child age (months)** |  |  |  |  |
| 6-11 |  | Ref |  | Ref |
| 12-23 |  | 0.93(0.61-1.42) |  | 1.02 (0.72,1.45) |
| 24-35 |  | 0.44(0.29-0.68)*** |  | 0.39 (0.28-0.55)*** |
| 36-47 |  | 0.28(0.18-0.43)*** |  | 0.26 (0.18-0.37)*** |
| 48-59 |  | 0.21(0.14-0.34)*** |  | 0.21 (0.14-0.29*** |
| **Recent fever** |  |  |  |  |
| No |  | Ref |  | Ref |
| Yes |  | 1.05(0.80-1.36)* |  | 1.08 (0.88-1.34)* |
| **Recent diarrhea** |  |  |  |  |
| No |  | Ref |  | Ref |
| Yes |  | 0.97(0.63-1.49)* |  | 0.69 (0.53-0.89) |
| **Children stunted** |  |  |  |  |
| Not stunted |  | Ref |  | NA |
| Stunted |  | 1.27(0.97-1.65) |  | NA |
| **Children underweight** |  |  |  |  |
| Not underweight |  | Ref |  | NA |
| Underweight |  | 1.18(0.87-1.60) |  | NA |
| **Children wasted** |  |  |  |  |
| Not wasted |  | Ref |  | NA |
| Wasted |  | 1.26(0.69-1.46) |  | NA |
| **Mothers age (years)** |  |  |  |  |
| 15-24 |  | Ref |  | Ref |
| 25-34 |  | 0.79 (0.62-1.01) |  | 0.79 (0.62-1.01) |
| 35-49 |  | 0.76 (0.58-0.99)* |  | 0.78 (0.59-0.99)* |
| **Mothers education level** |  |  |  |  |
| No education |  | Ref |  | Ref |
| Primary |  | 1.19 (0.92-1.54)* |  | 1.19 (0.92,1.53) |
| Secondary or above |  | 1.04 (0.77-1.39) |  | 1.02 (0.76-1.38) |
| **Wealth quintile** |  |  |  |  |
| Poorest |  | Ref |  | Ref |
| Poor |  | 0.92 (0.72-1.17) |  | 0.91 (0.72-1.15) |
| Middle |  | 0.87 (0.67-1.13) |  | 0.87 (0.66-1.16) |
| Richer |  | 0.76 (0.57-1.01)* |  | 0.77 (0.57-1.05) |
| Richest |  | 0.93 (0.66-1.31) |  | 0.90 (0.62-1.30) |
| **Maternal anemia** |  |  |  |  |
| Not anemic |  | Ref |  | Ref |
| Anemic |  | 2.07(1.66-2.56)*** |  | 1.59 (1.34-1.91)*** |
| **Community-level factors** |  |  |  |  |
| **Place of residence** |  |  |  |  |
| Urban |  |  | Ref | NA |
| Rural |  |  | 0.82 (0.63-1.08) | NA |
| **Community maternal anemia** |  |  |  |  |
| Low |  |  | Ref | Ref |
| High |  |  | 1.37 (1.14-1.65)** | 1.33 (1.08-1.64)** |
| **Community parity** |  |  |  |  |
| Low |  |  | Ref | Ref |
| High |  |  | 0.76 (0.62-0.93)*** | 0.89 (0.72-1.11) |
| **Community wealth** |  |  |  |  |
| Low |  |  | Ref | Ref |
| High |  |  | 1.09 (0.88-1.35)* | 1.04 (0.81-1.35) |
| **Community female education** |  |  |  |  |
| Low |  |  | Ref | Ref |
| High |  |  | 0.70 (0.54-0.90)** | 0.96 (0.81-1.35) |
| **Community safe water access** |  |  |  |  |
| Low |  |  | Ref | NA |
| High |  |  | 1.01 (0.83-1.23) |  |
| **Community toilet facility** |  |  |  |  |
| Low |  |  | Ref | NA |
| High |  |  | 1.09 (0.83-1.23) |  |
| **Random effect** |  |  |  |  |
| Community-level variance (SE) | 0.312(0.072)*** | 0.325(0.080)*** | 0.096(0.066)* | 0.293 (0.076)*** |
| ICC (%) | 8.52% | 8.99% | 2.84% | 8.2 |
| PCV (%) | Reference | 4% | 33% | 4 |
| MOR |  |  |  |  |
| **Model fit statistics** |  |  |  |  |
| AIC | 3988.04 | 3643.11 | 2335.76 | 3713.4 |
| BIC | 4000.00 | 3768.39 | 2348.68 | 3839.1 |

**Nepal**

| **Individual-and community-level characteristics** | **Model 1**  **Empty model** | **Model 2**  **Individual-level variable** | **Model 3**  **Community level variable** | **Model 4**  **Individual- and community-level variable** |
| --- | --- | --- | --- | --- |
| **Individual level** |  |  |  |  |
| **Sex of child** |  |  |  |  |
| Male |  | Ref |  | NA |
| Female |  | 1.03(0.83-1.27) |  | NA |
| **Child age (months)** |  |  |  |  |
| 6-11 |  | Ref |  | Ref |
| 12-23 |  | 0.93(0.61-1.42) |  | 0.95 (0.62-1.47) |
| 24-35 |  | 0.44(0.29-0.68)*** |  | 0.45 (0.29-0.69)*** |
| 36-47 |  | 0.28(0.18-0.43)*** |  | 0.28 (0.14-0.34)*** |
| 48-59 |  | 0.21(0.14-0.34)*** |  | 0.22 (0.14-0.34)*** |
| **Recent fever** |  |  |  |  |
| No |  | Ref |  | NA |
| Yes |  | 1.05(0.80-1.36) |  | NA |
| **Recent diarrhea** |  |  |  |  |
| No |  | Ref |  | NA |
| Yes |  | 0.97(0.63-1.49) |  | NA |
| **Children stunted** |  |  |  |  |
| Not stunted |  | Ref |  | Ref |
| Stunted |  | 1.27(0.97-1.65)* |  | 1.27 (0.97-1.67)* |
| **Children underweight** |  |  |  |  |
| Not underweight |  | Ref |  | Ref |
| Underweight |  | 1.18(0.87-1.60)* |  | 1.18 (0.87-1.59) |
| **Children wasted** |  |  |  |  |
| Not wasted |  | Ref |  | Ref |
| Wasted |  | 1.26(0.84-1.90)* |  | 1.27 (0.83-1.90) |
| **Mothers age (years)** |  |  |  |  |
| 15-24 |  | Ref |  | Ref |
| 25-34 |  | 0.96 (0.75-1.22)* |  | 0.98 (0.77-1.24) |
| 35-49 |  | 0.78 (0.52-1.16) |  | 0.77 (0.51-1.15) |
| **Mothers education level** |  |  |  |  |
| No education |  | Ref |  | Ref |
| Primary |  | 0.73 (0.53-1.01) |  | 0.75 (0.54-1.04) |
| Secondary or above |  | 0.61 (0.46-0.81)*** |  | 0.68 (0.50-0.93)* |
| **Wealth quintile** |  |  |  |  |
| Poorest |  | Ref |  | Ref |
| Poor |  | 0.88 (0.64-1.21) |  | 0.76 (0.55-1.01) |
| Middle |  | 1.39 (1.00-1.95)* |  | 1.05 (0.71-1.59) |
| Richer |  | 1.24 (0.88-1.75) |  | 0.91 (0.58-1.42) |
| Richest |  | 0.79 (0.52-1.20) |  | 0.55 (0.32-0.94)* |
| **Maternal anemia** |  |  |  |  |
| Not anemic |  | Ref |  | Ref |
| Anemic |  | 2.07(1.66-2.56)*** |  | 1.71 (1.36-2.15)*** |
| **Community-level factors** |  |  |  |  |
| **Place of residence** |  |  |  |  |
| Urban |  |  | Ref | Ref |
| Rural |  |  | 1.26 (0.98-1.62)* | 1.08 (0.84-1.39) |
| **Community maternal anemia** |  |  |  |  |
| Low |  |  | Ref | Ref |
| High |  |  | 1.42 (0.94-2.15)* | 1.72 (1.34-2.23)*** |
| **Community parity** |  |  |  |  |
| Low |  |  | Ref | Ref |
| High |  |  | 1.05 (0.81-1.36)* | 0.97 (0.74-1.27) |
| **Community wealth** |  |  |  |  |
| Low |  |  | Ref | Ref |
| High |  |  | 0.72 (0.55-0.93)* | 0.78 (0.55-1.10)* |
| **Community female education** |  |  |  |  |
| Low |  |  | Ref | Ref |
| High |  |  | 0.82 (0.62-1.08) | 1.02 (0.75-1.39) |
| **Community safe water access** |  |  |  |  |
| Low |  |  | Ref | Ref |
| High |  |  | 0.96 (0.74-1.24) | 1.01(0.77-1.31) |
| **Community toilet facility** |  |  |  |  |
| Low |  |  | Ref | Ref |
| High |  |  | 1.30 (0.97-1.75) | 1.05 (0.78-1.42) |
| **Random effect** |  |  |  |  |
| Community-level variance (SE) | 0.355(0.110)*** | 0.194(0.101)** | 0.270(0.098)*** | 0.156 (0.095)* |
| ICC (%) | 9.74% | 5.64% | 7.66% | 4.4% |
| PCV (%) | Reference | 46% | 24% | 56% |
| **Model fit statistics** |  |  |  |  |
| AIC | 2404.461 | 2199.14 | 2392.81 | 2193.5 |
| BIC | 2415.39 | 2313.74 | 2441.99 | 2330 |
