# Supplementary material for: Factors associated with anemia among children in South and Southeast Asia: a multilevel analysis
Source: BMC Public Health. 2023 Feb 15;23:343. doi: 10.1186/s12889-023-15265-y (PMC9933407; doi:10.1186/s12889-023-15265-y)
Supplement: Supplementary file 3 — Supplementary Material 3 [file 12889_2023_15265_MOESM3_ESM.docx]

**S3 Table. VIF for the six selected South and Southeast Asian countries**

| Variables | Bangladesh | Cambodia | India | Maldives | Myanmar | Nepal |
| --- | --- | --- | --- | --- | --- | --- |
|  | VIF | VIF | VIF | VIF | VIF | VIF |
| **Individual-level factors** |  |  |  |  |  |  |
| Sex of child | 1.01 | 1.00 | 1.00 | 1.02 | 1.01 | 1.01 |
| Child age | 1.09 | 1.09 | 1.10 | 1.07 | 1.09 | 1.12 |
| Recent fever | 1.03 | 1.10 | 1.07 | 1.08 | 1.07 | 1.08 |
| Recent diarrhea | 1.01 | 1.12 | 1.08 | 1.06 | 1.08 | 1.08 |
| Children stunted | 1.52 | 1.43 | 1.45 | 1.31 | 1.37 | 1.51 |
| Children underweight | 1.94 | 1.66 | 1.74 | 1.70 | 1.52 | 1.71 |
| Children wasted | 1.35 | 1.22 | 1.34 | 1.40 | 1.21 | 1.25 |
| Mother age | 1.15 | 1.10 | 1.13 | 1.30 | 1.09 | 1.22 |
| Mother education level | 1.43 | 1.30 | 1.71 | 1.40 | 1.48 | 1.64 |
| Wealth quintile | 1.33 | 1.32 | 2.38 | 1.61 | 2.02 | 2.26 |
| Maternal anemia | 1.02 | 1.02 | 1.21 | 1.13 | 1.14 | 1.11 |
| **Community-level factors** |  |  |  |  |  |  |
| Place of residence | 1.32 | 1.62 | 1.40 | 1.45 | 1.66 | 1.21 |
| Community maternal anemia^a^ | 1.31 | 1.03 | 1.23 | 1.15 | 1.18 | 1.10 |
| Community parity^b^ | 1.20 | 1.14 | 1.26 | 1.18 | 1.16 | 1.29 |
| Community wealth^c^ | 1.57 | 1.44 | 2.00 | 1.37 | 1.73 | 2.21 |
| Community female education^d^ | 1.47 | 1.33 | 1.76 | 1.29 | 1.59 | 1.72 |
| Community safe water access^e^ | 1.05 | 1.25 | 1.02 | 1.14 | 1.07 | 1.13 |
| Community toilet facility^f^ | 1.13 | 1.66 | 1.55 | 1.04 | 1.21 | 1.41 |

**Note:** VIF: Variance Inflation Factor; amean percent of women with Hb levels less than 12 g/dL; ^b^ mean percent of women with fertility rate of 5 children and above; ^c^ mean percent of households wealth quintiles categorized richer and richest and above; ^d^ mean percent of women with primary education level and above; ^e^mean percent of households with access to improved water source (piped water into dwelling, piped water to yard/plot, public tap or standpipe, tube well or borehole, protected dug well protected spring and rainwater) ^f^ mean percent of household with access to improved toilet facility (flush toilet, piped sewer system, septic tank, flush/pour flush to pit latrine, ventilated improved pit latrine, pit latrine with slab, and composting toilet)
